# Supplementary material for: Characteristics and dynamical signatures of recurrent cortical circuits during context-dependent processing
Source: bioRxiv. 2026 Feb 6:2026.02.06.704473. Preprint. [Version 1] doi: 10.64898/2026.02.06.704473 (PMC12889673; doi:10.64898/2026.02.06.704473)
Supplement: 1 [file NIHPP2026.02.06.704473V1-supplement-1.pdf]

bioRxiv preprint doi: https://doi.org/10.64898/2026.02.06.704473; this version posted February 6, 2026. The copyright holder for this preprint (which was not certified by peer review) is the author/funder, who has granted bioRxiv a license to display the preprint in perpetuity. It is made available under aCC-BY 4.0 International license.

Supplementary Figures

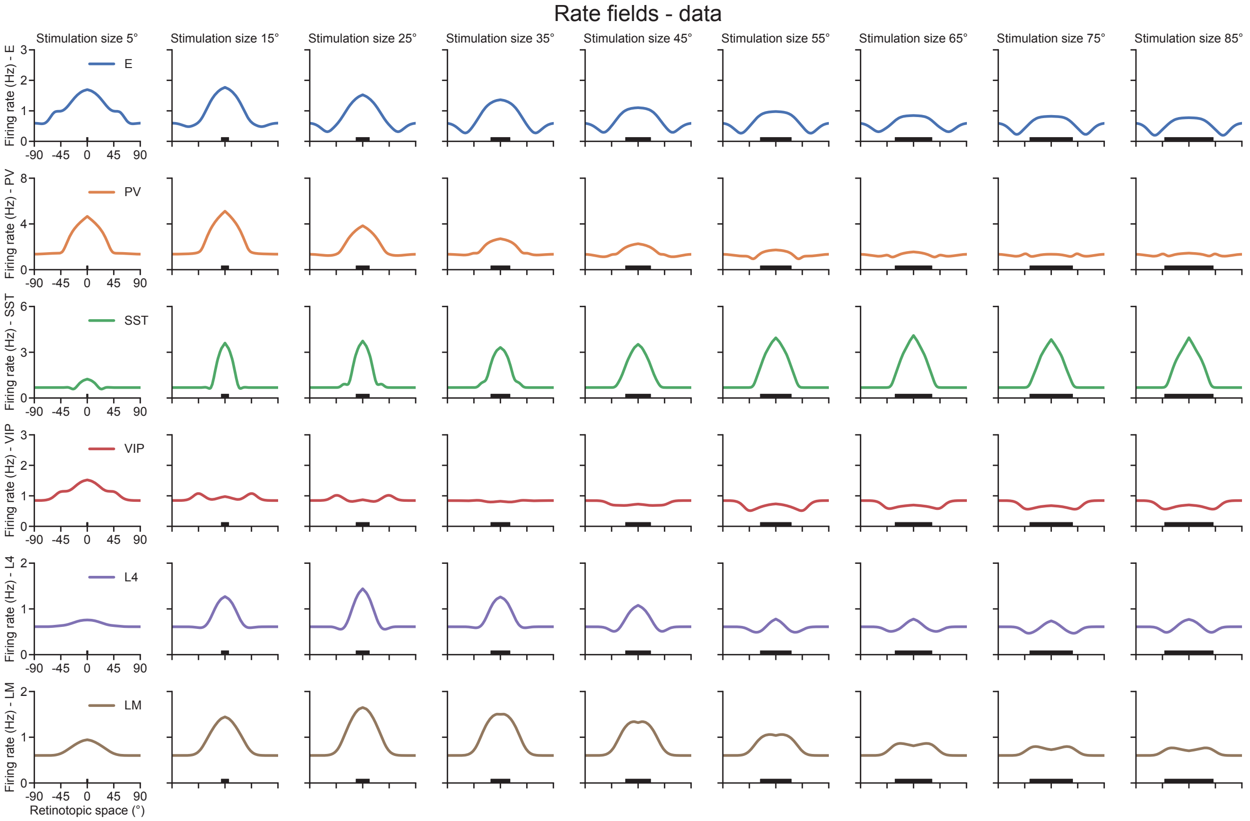

Fig. S1. Rate field data of E, PV, SST, VIP, L4, and LM populations across stimulus sizes ranging from 5° to 85°. Black bars indicate stimulus size. Adapted from Di Santo et al., 2025.

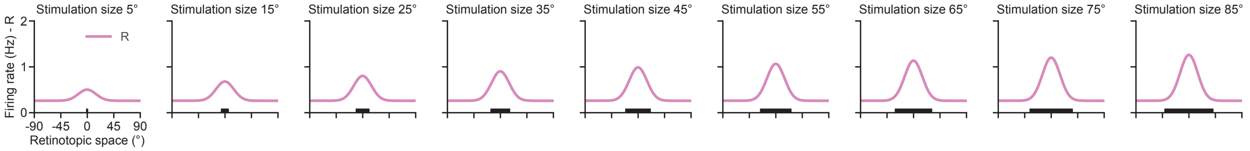

Fig. S2. Constructed rate fields of the residual input across stimulus sizes. The peak amplitude of the rate field increases with stimulus size, while its spatial scale σ_R (see Eq. 8) remains fixed at 15° across all stimulus conditions.

bioRxiv preprint doi: https://doi.org/10.64898/2026.02.06.704473; this version posted February 6, 2026. The copyright holder for this preprint (which was not certified by peer review) is the author/funder, who has granted bioRxiv a license to display the preprint in perpetuity. It is made available under aCC-BY 4.0 International license.

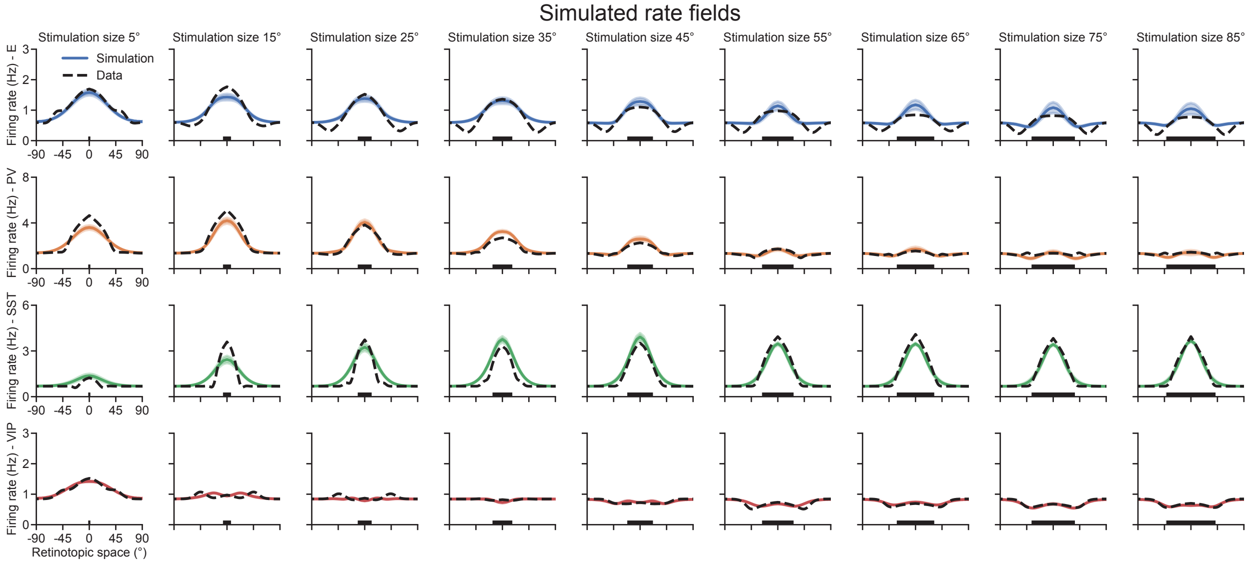

Fig. S3. Simulated rate fields of E, PV, SST, VIP populations across stimulus sizes from the top ten optimized models. Solid lines and shaded regions indicate the mean and the standard deviation of the simulated rate fields, respectively. Dashed lines represent the corresponding data.

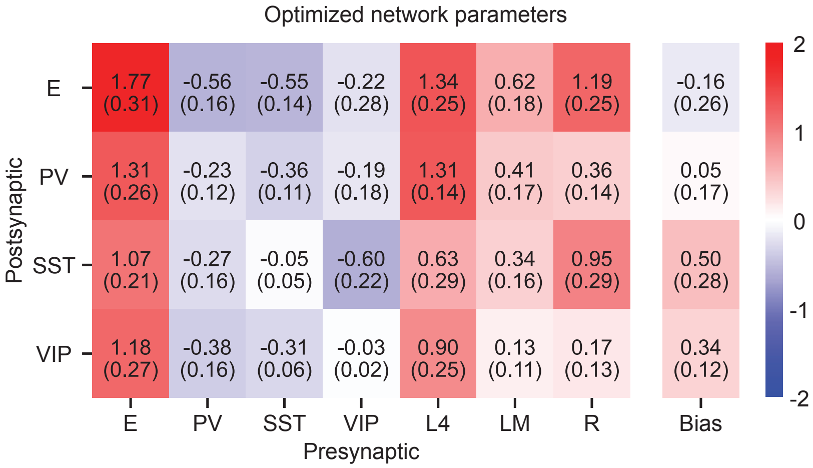

Fig. S4. Connection strengths and biases from the top ten optimized models. The mean values and standard deviations are shown above and below, respectively. Color codes the mean value.

bioRxiv preprint doi: https://doi.org/10.64898/2026.02.06.704473; this version posted February 6, 2026. The copyright holder for this preprint (which was not certified by peer review) is the author/funder, who has granted bioRxiv a license to display the preprint in perpetuity. It is made available under aCC-BY 4.0 International license.

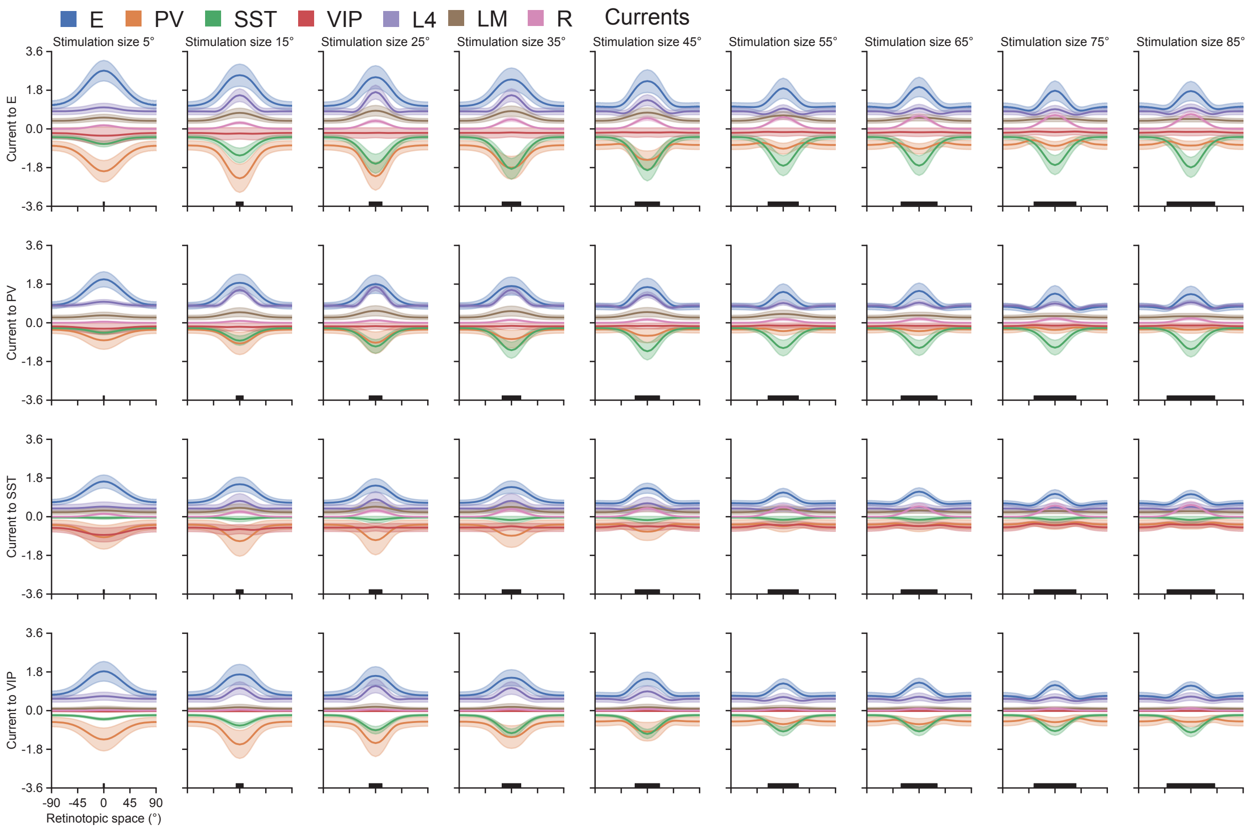

Fig. S5. Different current sources to E, PV, SST, and VIP populations across stimulus sizes. Solid lines and shaded regions indicate the mean and the standard deviation, respectively, of the corresponding currents from the top ten optimized models.

bioRxiv preprint doi: https://doi.org/10.64898/2026.02.06.704473; this version posted February 6, 2026. The copyright holder for this preprint (which was not certified by peer review) is the author/funder, who has granted bioRxiv a license to display the preprint in perpetuity. It is made available under aCC-BY 4.0 International license.

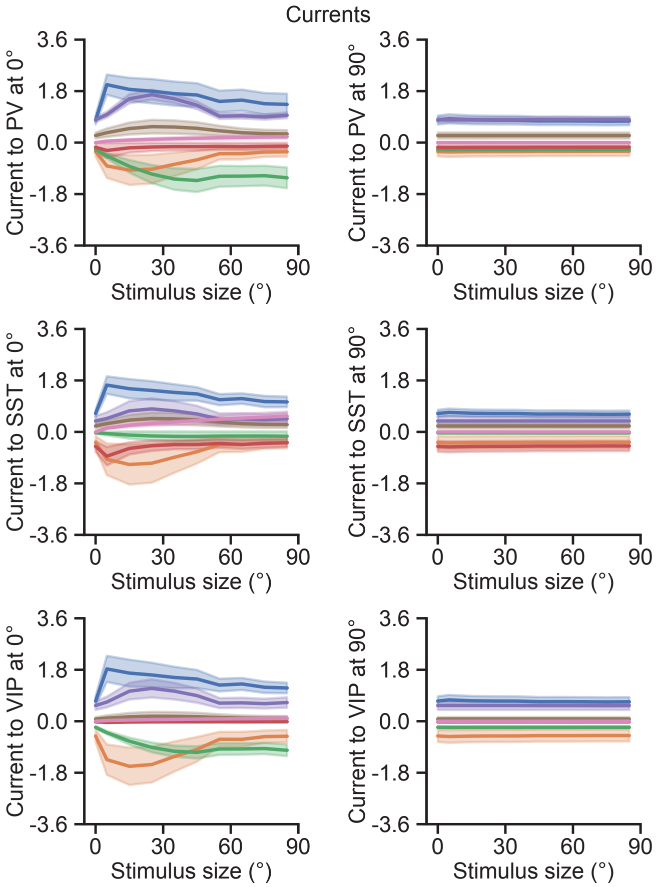

Fig. S6. Left: Current from different sources to different types of inhibitory neurons (top: PV, middle: SST, bottom: VIP) located at 0° in the retinotopic space as a function of stimulus size. Solid lines and shaded regions indicate the mean and the standard deviation of the corresponding currents, respectively. Right: Same as left but for different types of inhibitory neurons located at 90° in the retinotopic space.

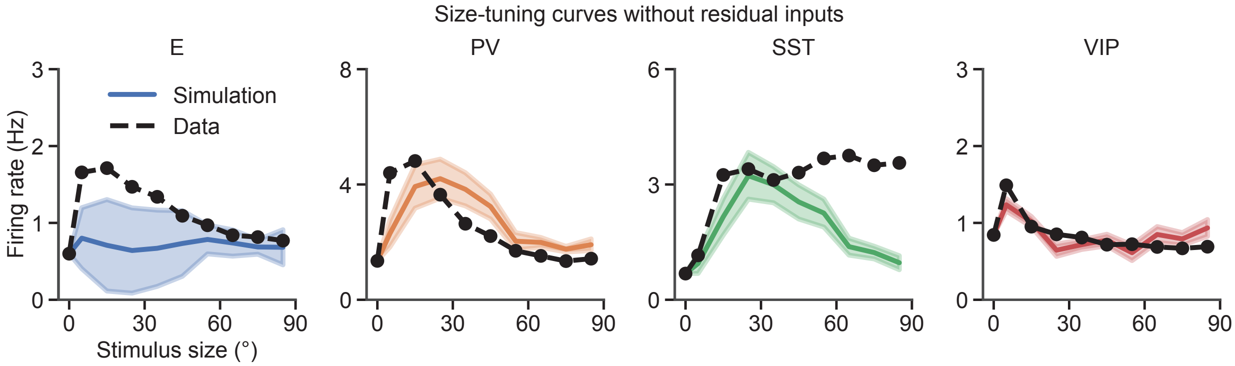

Fig. S7. Size-tuning curves of E, PV, SST, and VIP populations from the top ten models obtained by optimizing without residual inputs. Solid lines and shaded regions indicate the mean and the standard deviation of the size-tuning curves, respectively. Dashed lines represent the corresponding data.

bioRxiv preprint doi: https://doi.org/10.64898/2026.02.06.704473; this version posted February 6, 2026. The copyright holder for this preprint (which was not certified by peer review) is the author/funder, who has granted bioRxiv a license to display the preprint in perpetuity. It is made available under aCC-BY 4.0 International license.

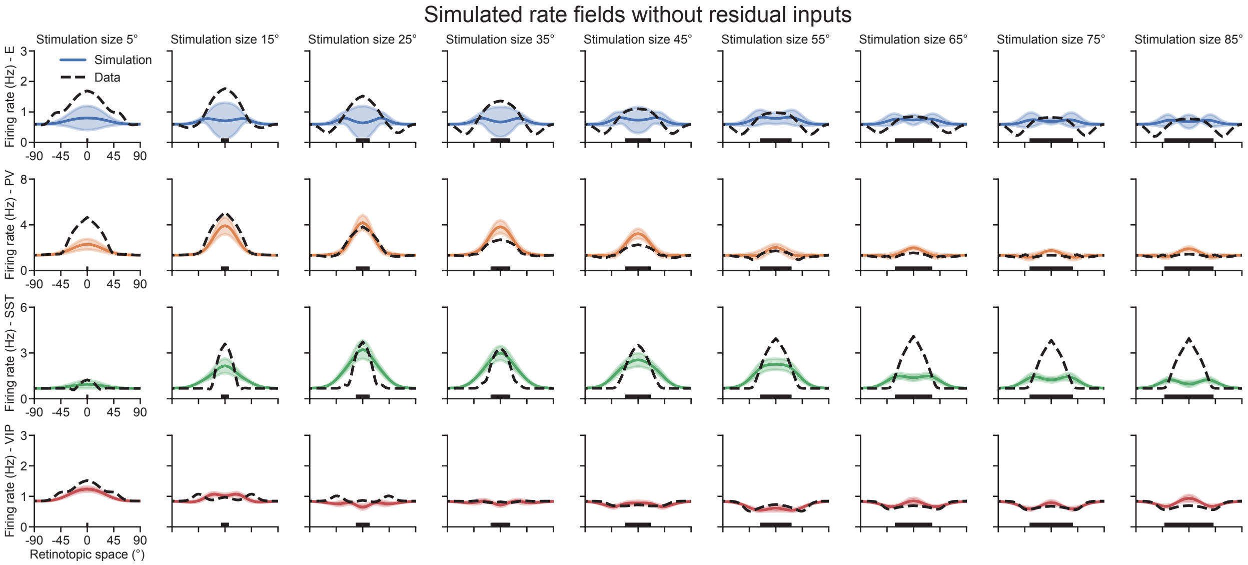

Fig. S8. Simulated rate fields of E, PV, SST, VIP populations across stimulus sizes from the top ten models obtained by optimizing without residual inputs. Solid lines and shaded regions indicate the mean and the standard deviation of the simulated rate fields, respectively. Dashed lines represent the corresponding data.

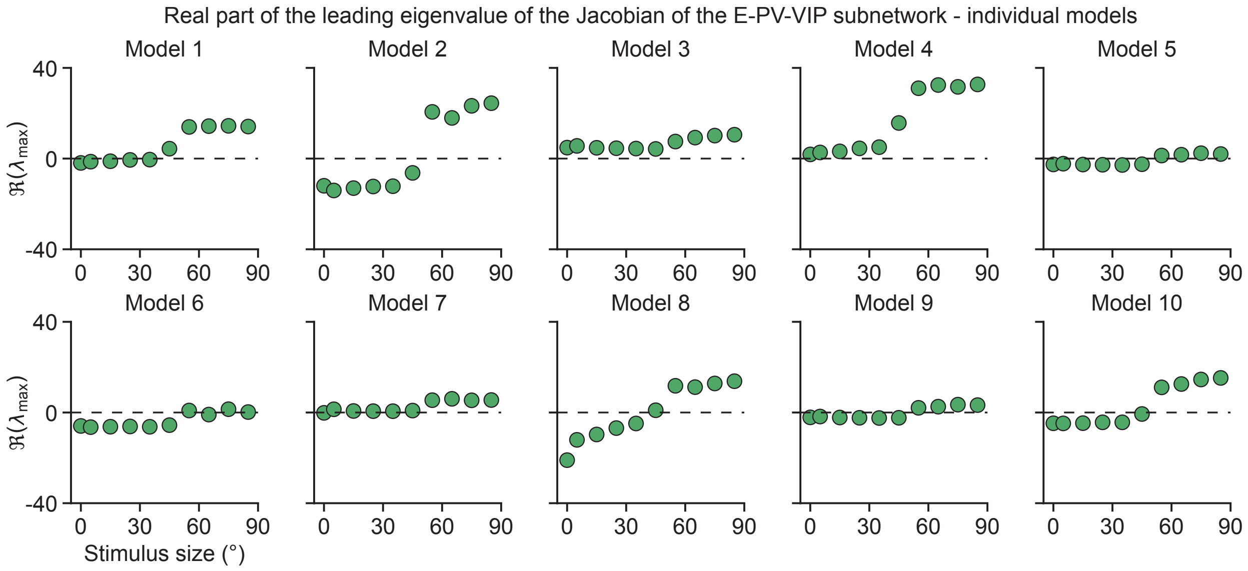

Fig. S9. Real part of the leading eigenvalue of the Jacobian of the E-PV-VIP subnetwork as a function of stimulus size. Each subplot corresponds to one model.

bioRxiv preprint doi: https://doi.org/10.64898/2026.02.06.704473; this version posted February 6, 2026. The copyright holder for this preprint (which was not certified by peer review) is the author/funder, who has granted bioRxiv a license to display the preprint in perpetuity. It is made available under aCC-BY 4.0 International license.

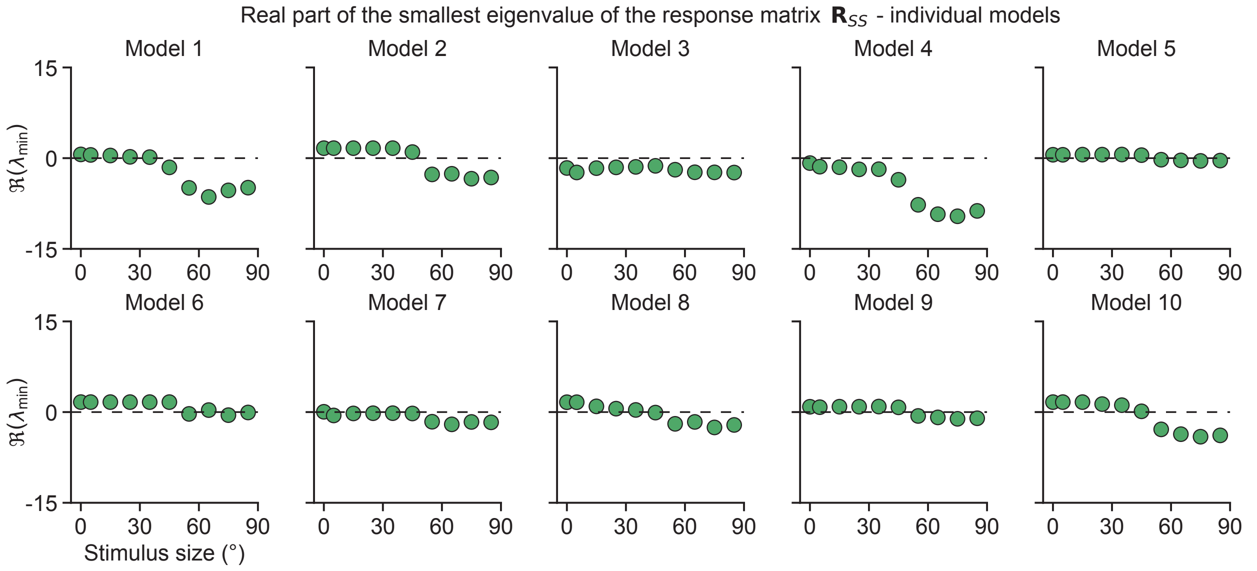

Fig. S10. Real part of the smallest eigenvalue of the response matrix $\mathbf{R}_{SS}$ as a function of stimulus size. Each subplot corresponds to one model.

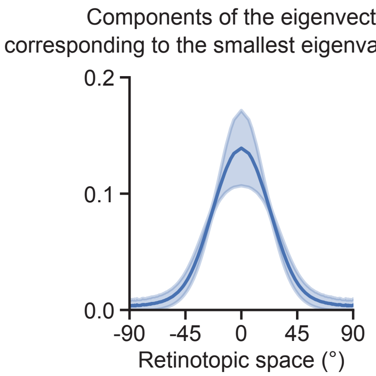

Fig. S11. Components of the eigenvector corresponding to the smallest eigenvalue of the response matrix $\mathbf{R}_{SS}$ as a function of retinotopic space. Solid lines and shaded regions indicate the corresponding mean and the standard deviation. Eigenvectors are computed at a stimulus size of 55°, where the real parts of the smallest eigenvalues of the response matrix $\mathbf{R}_{SS}$ are negative for all ten models (Fig. S10).

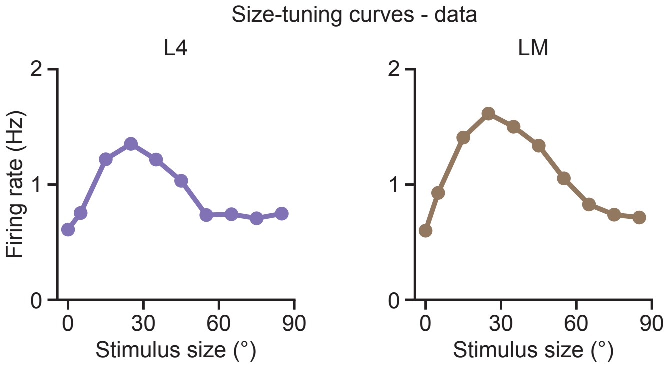

Fig. S12. Size-tuning curve data of L4 (left) and LM (right).

bioRxiv preprint doi: https://doi.org/10.64898/2026.02.06.704473; this version posted February 6, 2026. The copyright holder for this preprint (which was not certified by peer review) is the author/funder, who has granted bioRxiv a license to display the preprint in perpetuity. It is made available under aCC-BY 4.0 International license.

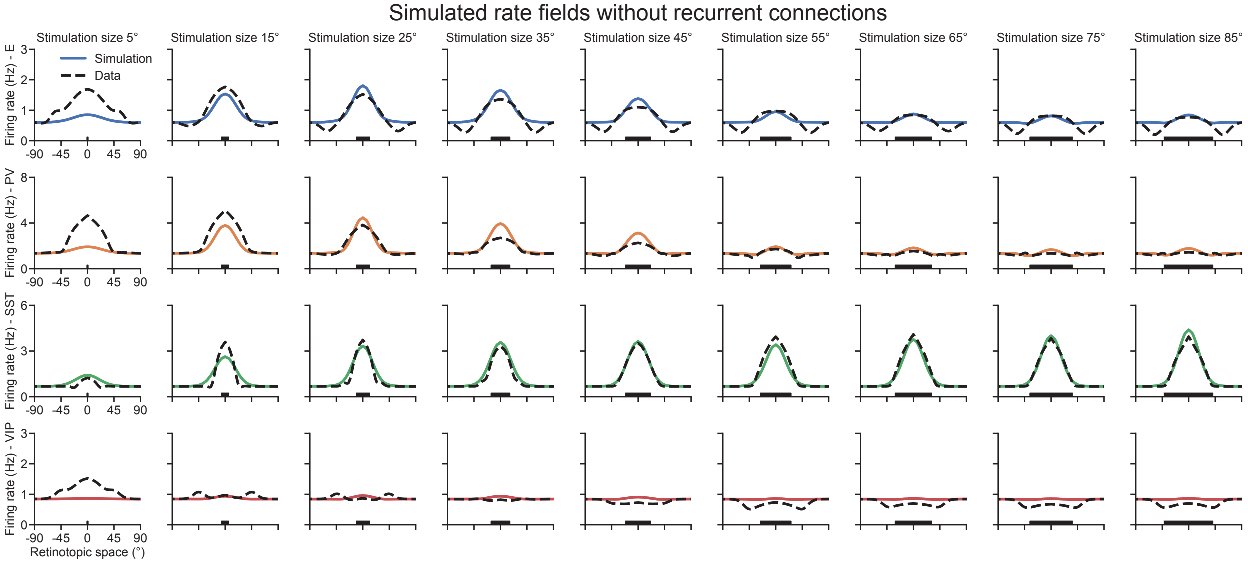

Fig. S13. Simulated rate fields of E, PV, SST, and VIP populations across stimulus sizes from the top ten models obtained by optimizing without recurrent connections.

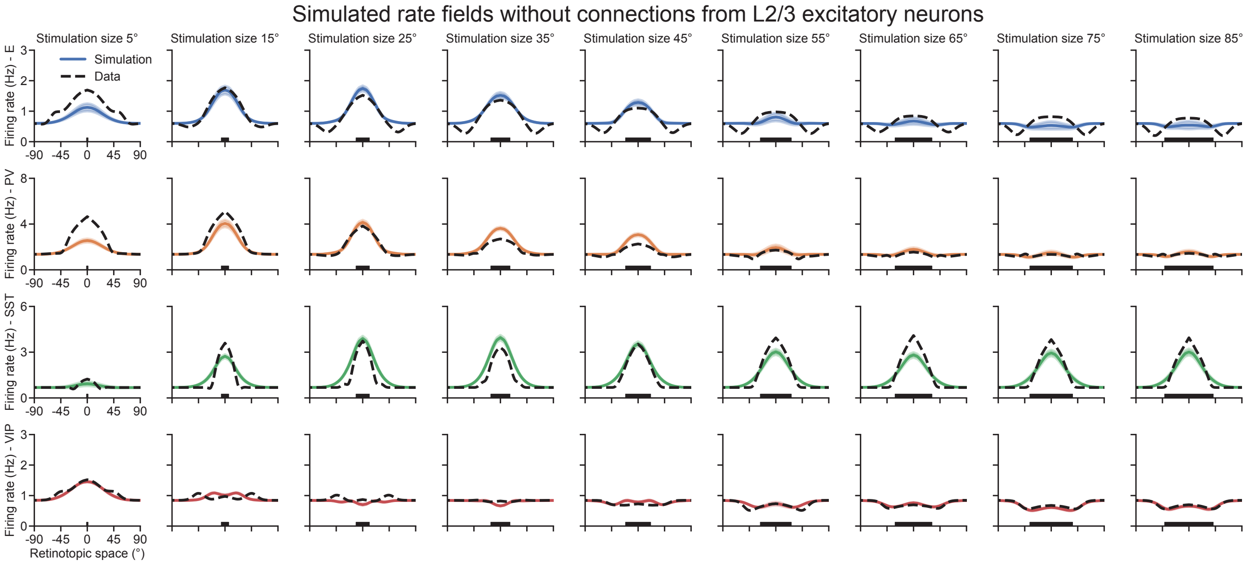

Fig. S14. Simulated rate fields of E, PV, SST, and VIP populations across stimulus sizes from the top ten models obtained by optimizing without connections from L2/3 excitatory neurons. Solid lines and shaded regions indicate the mean and the standard deviation of the simulated rate fields, respectively. Dashed lines represent the corresponding data.

bioRxiv preprint doi: https://doi.org/10.64898/2026.02.06.704473; this version posted February 6, 2026. The copyright holder for this preprint (which was not certified by peer review) is the author/funder, who has granted bioRxiv a license to display the preprint in perpetuity. It is made available under aCC-BY 4.0 International license.

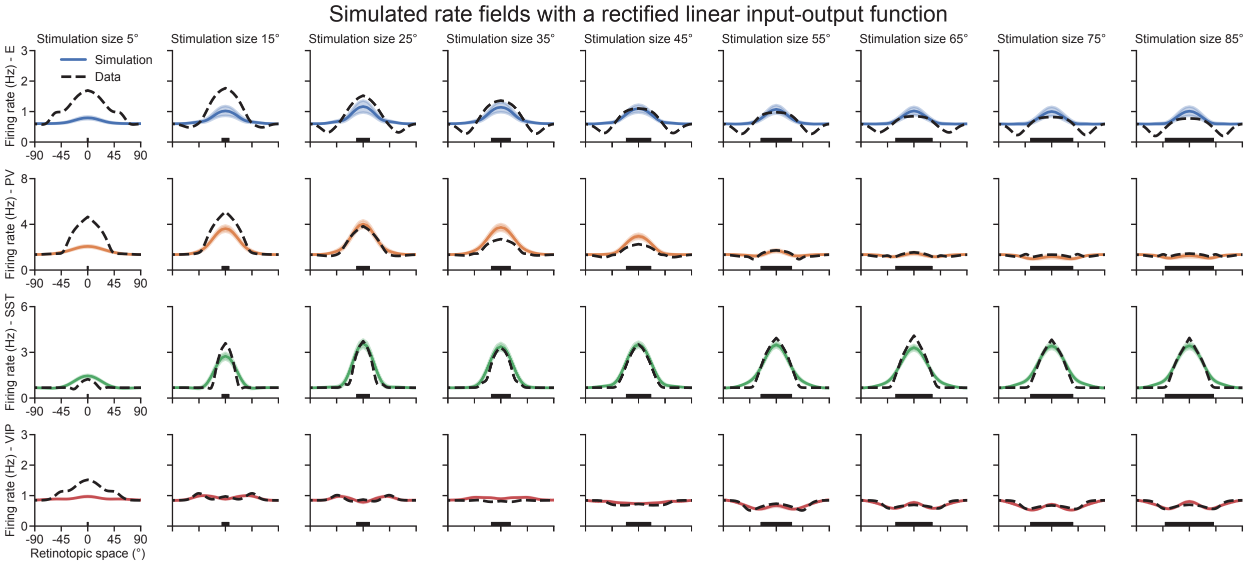

Fig. S15. Simulated rate fields of E, PV, SST, and VIP populations across stimulus sizes from the top ten models obtained by optimizing using a rectified linear rather than rectified quadratic input-output function. Solid lines and shaded regions indicate the mean and the standard deviation of the simulated rate fields, respectively. Dashed lines represent the corresponding data.

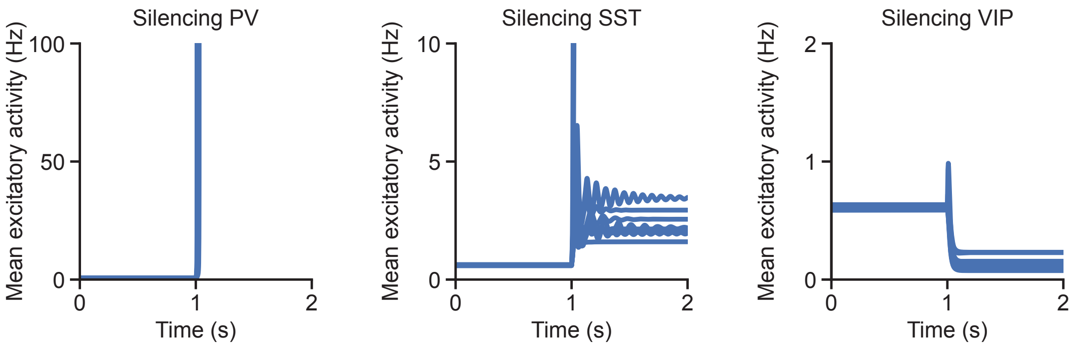

Fig. S16. Left: Mean excitatory activity following PV silencing at 1 s for stimulus size 55°. Each line corresponds to one model. In all ten models, excitatory activity exhibits runaway dynamics after PV silencing. Middle: Same as left, but with SST silencing. In eight out of ten models, excitatory activity remains within physiologically realistic levels after SST silencing. Right: Same as left, but with VIP silencing.

bioRxiv preprint doi: https://doi.org/10.64898/2026.02.06.704473; this version posted February 6, 2026. The copyright holder for this preprint (which was not certified by peer review) is the author/funder, who has granted bioRxiv a license to display the preprint in perpetuity. It is made available under aCC-BY 4.0 International license.

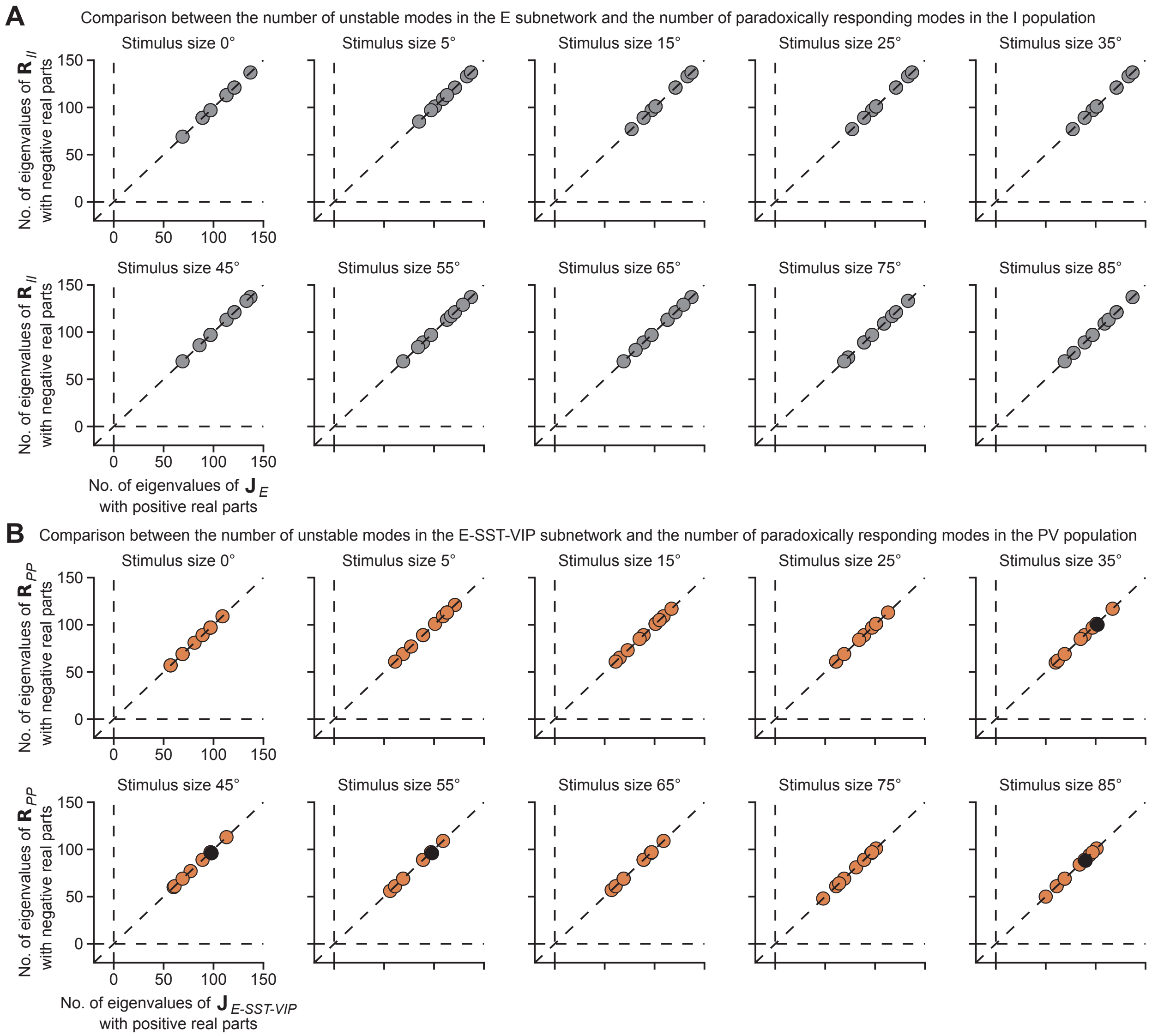

Fig. S17. A. Comparison between the number of unstable modes in the E subnetwork and the number of paradoxically responding modes in the I population. The number of unstable modes in the E subnetwork is quantified by counting the eigenvalues of the Jacobian of the E subnetwork $\mathbf{J}_E$ with positive real parts shown on the x-axis. The number of paradoxically responding modes in the I population is quantified by counting the eigenvalues of the corresponding response matrix $\mathbf{R}_{II}$ with negative real parts shown on the y-axis. Results for different stimulus sizes are shown in separate panels. Each dot represents one of the ten models. All dots have equal, positive x and y values and lie along the diagonal lines, indicating that the parity of the number of unstable modes in the E subnetwork matches the parity of the number of paradoxically responding modes in the I population. Note that some dots are located at the same coordinates. **B.** Same as A, but for the comparison between the number of unstable models in the E-SST-VIP network and the number of paradoxically responding modes in the PV population. The numbers are quantified by the eigenvalues of the Jacobian of the E-SST-VIP subnetwork $\mathbf{J}_{E-SST-VIP}$ with positive real parts shown on the x-axis and the eigenvalues of the corresponding response matrix $\mathbf{R}_{PP}$ with negative real parts shown on the y-axis, respectively. Some dots do not lie exactly on the diagonal and are colored black (corresponding to x-y pairs of 102-100 at a stimulus size of 35°, two points at 98-96 for 45°, one point at 98-96 for 55°, and one point at 90-88 for 85°), indicating that despite having the same parity, these two values need not be identical.

bioRxiv preprint doi: https://doi.org/10.64898/2026.02.06.704473; this version posted February 6, 2026. The copyright holder for this preprint (which was not certified by peer review) is the author/funder, who has granted bioRxiv a license to display the preprint in perpetuity. It is made available under aCC-BY 4.0 International license.

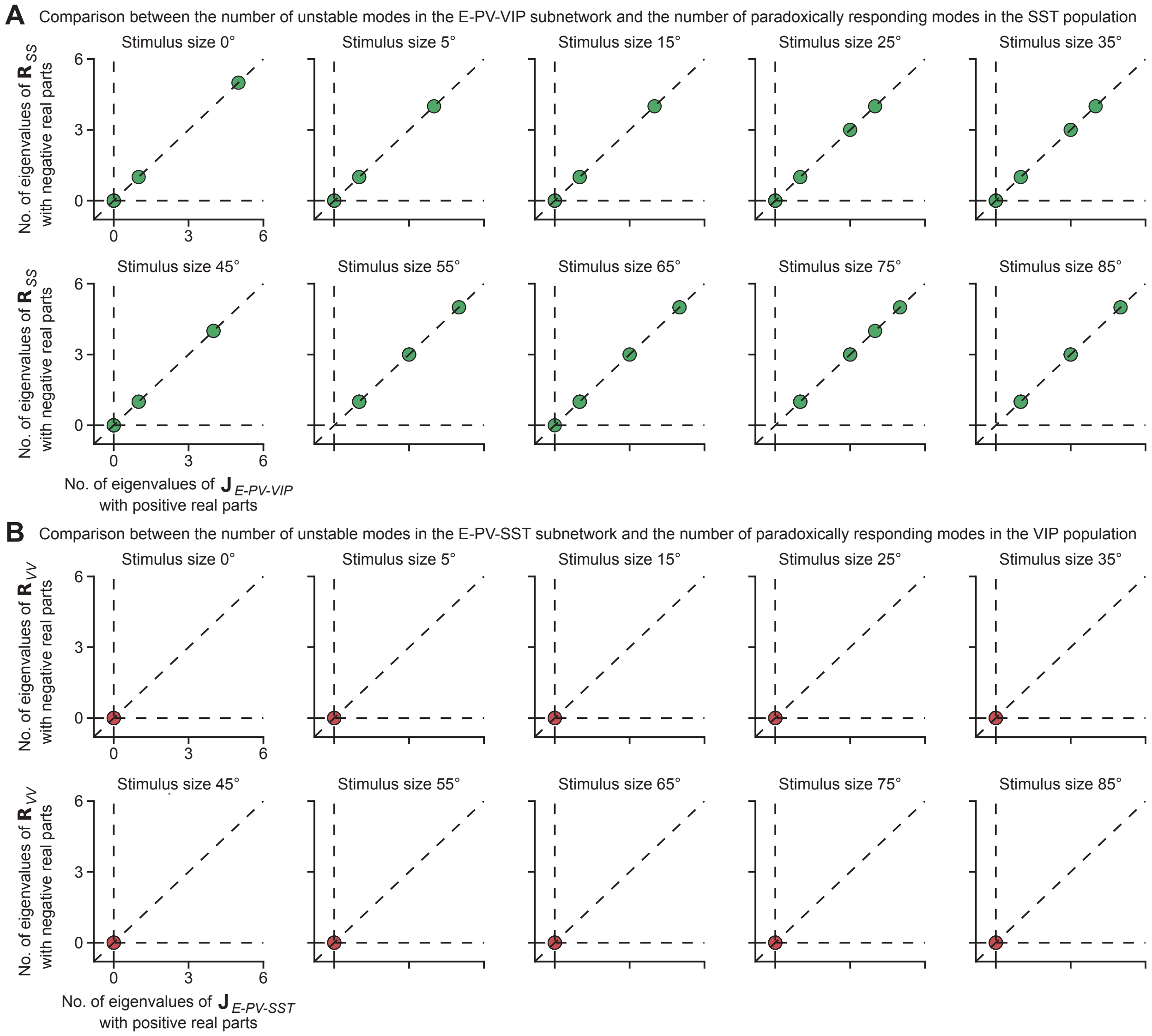

Fig. S18. A. Comparison between the number of unstable models in the E-PV-VIP subnetwork and the number of paradoxically responding modes in the SST population. The number of unstable modes in the E-PV-VIP subnetwork is quantified by counting the eigenvalues of the Jacobian of the E-PV-VIP subnetwork $\mathbf{J}_{E-PV-VIP}$ with positive real parts shown on the x-axis. The number of paradoxically responding modes in the SST population is quantified by counting the eigenvalues of the corresponding response matrix $\mathbf{R}_{SS}$ with negative real parts shown on the y-axis. Results for different stimulus sizes are shown in separate panels. Each dot represents one of the ten models. All dots lie along the diagonal lines, indicating that the parity of the number of unstable modes in the E-PV-VIP subnetwork matches the parity of the number of paradoxically responding modes in the SST population. Note that some dots are located at the same coordinates. **B.** Same as A, but for the comparison between the number of unstable models in the E-PV-SST network and the number of paradoxically responding modes in the VIP population. The numbers are quantified by the eigenvalues of the Jacobian of the E-PV-SST subnetwork $\mathbf{J}_{E-PV-SST}$ with positive real parts shown on the x-axis and the eigenvalues of the corresponding response matrix $\mathbf{R}_{VV}$ with negative real parts shown on the y-axis, respectively. All dots are located at the origin, implying that VIP neurons are not required for stabilization and that patterned perturbations do not elicit paradoxical responses in the VIP population.

bioRxiv preprint doi: https://doi.org/10.64898/2026.02.06.704473; this version posted February 6, 2026. The copyright holder for this preprint (which was not certified by peer review) is the author/funder, who has granted bioRxiv a license to display the preprint in perpetuity. It is made available under aCC-BY 4.0 International license.

Table S1: Parameters.

**Symbol**	**Value**	**Unit**	**Description**
σ_{EE}	7	degree	spatial scale of the Gaussian connectivity profile for E to E connections
σ_{EP}	5	degree	spatial scale of the Gaussian connectivity profile for PV to E connections
σ_{ES}	7	degree	spatial scale of the Gaussian connectivity profile for SST to E connections
σ_{EV}	5	degree	spatial scale of the Gaussian connectivity profile for VIP to E connections
σ_{PE}	5	degree	spatial scale of the Gaussian connectivity profile for E to PV connections
σ_{PP}	4	degree	spatial scale of the Gaussian connectivity profile for PV to PV connections
σ_{PS}	5	degree	spatial scale of the Gaussian connectivity profile for SST to PV connections
σ_{PV}	4	degree	spatial scale of the Gaussian connectivity profile for VIP to PV connections
σ_{SE}	7	degree	spatial scale of the Gaussian connectivity profile for E to SST connections
σ_{SP}	5	degree	spatial scale of the Gaussian connectivity profile for PV to SST connections
σ_{SS}	7	degree	spatial scale of the Gaussian connectivity profile for SST to SST connections
σ_{SV}	5	degree	spatial scale of the Gaussian connectivity profile for VIP to SST connections
σ_{VE}	5	degree	spatial scale of the Gaussian connectivity profile for E to VIP connections
σ_{VP}	4	degree	spatial scale of the Gaussian connectivity profile for PV to VIP connections
σ_{VS}	7	degree	spatial scale of the Gaussian connectivity profile for SST to VIP connections
σ_{VV}	4	degree	spatial scale of the Gaussian connectivity profile for VIP to VIP connections
σ_{XL4}	7	degree	spatial scale of the Gaussian connectivity profile for connections from L4
σ_{XLM}	15	degree	spatial scale of the Gaussian connectivity profile for connections from LM
σ_{XR}	15	degree	spatial scale of the Gaussian connectivity profile for residual input connections
σ_{R}	15	degree	spatial scale of the residual input rate field
c_E	1	a.u.	optimization weighting factor for E responses
c_P	1	a.u.	optimization weighting factor for PV responses
c_S	1	a.u.	optimization weighting factor for SST responses
c_V	0.1	a.u.	optimization weighting factor for VIP responses
